# Public knowledge and awareness of osteoarthritis in Makkah and Jeddah: a comparative cross-sectional study

**DOI:** 10.1080/07853890.2026.2726697

**Published:** 2026-09-15

**Authors:** Ali H. Alyami, Abdalmalik T. Malki, Deemah M. Alharbi, Hasen H. Aljadani, Lameas A. Wali, Mahmoud A. Wazzan, Hamed W. Babtain, Awwab A. Shahhatalsayed, Abdulelah A. Alghamdi, Juman M. Almasaad

**Affiliations:** a College of Medicine, King Saud bin Abdulaziz University for Health Sciences, Jeddah, Saudi Arabia; b King Abdullah International Medical Research Center, Jeddah, Saudi Arabia; c Department of Orthopedic Surgery, Ministry of the National Guard-Health Affairs, Jeddah, Saudi Arabia

**Keywords:** Osteoarthritis, (awareness, health knowledge, attitudes, practice), Saudi Arabia, (cross-sectional studies), public health

## Abstract

**Background:**

Osteoarthritis (OA) is a major cause of disability, yet public awareness remains limited. This study assessed OA knowledge in Makkah and Jeddah and compared findings with previous Saudi studies.

**Methods:**

A comparative cross-sectional study was conducted from July to October 2025 among adults in Makkah and Jeddah. A self-administered online questionnaire assessed demographics, information sources, and OA knowledge (score: 0–20). Findings were compared with a 2020 Jeddah survey using overall and item-level knowledge and relevant subgroup scores. The study was IRB-approved (NRJ25/055/4). Analyses were performed using JMP Pro 19 (p ≤ 0.05).

**Results:**

Among 365 participants, 54.8% were male, and the mean knowledge score was 11.34 ± 3.48. Scores did not differ significantly by education (*p *= 0.51), age (*p* = 0.20), or gender (*p* = 0.97). Participants with a medical-field background had higher scores than those without (13.09 ± 2.74 vs. 10.88 ± 3.51; *p* < 0.0001). Most recognized aging as a risk factor (91.2%), physiotherapy as beneficial (82.5%), and multiple-joint involvement (81.9%), while misconceptions about cold weather, microorganisms, and acid-free diets persisted. Greater knowledge was associated with identifying cartilage degeneration, and school/university was associated with the highest awareness among information sources. Awareness showed modest improvement compared with previous regional studies, particularly regarding diagnosis and treatment.

**Conclusion:**

Public awareness of osteoarthritis in Makkah and Jeddah remains limited despite improvements compared with previous Saudi studies, highlighting the need for targeted education to address persistent misconceptions.

## Introduction

Osteoarthritis (OA) is a common degenerative joint disease characterized by increasing articular cartilage degeneration, resulting in pain, stiffness, and decreased mobility [1]. Osteoarthritis (OA) is a major cause of disability worldwide, particularly among older adults, ranking as the eleventh leading cause of disability globally [2–5]. The risk factors for OA are divided into two main categories: individual-level factors, including age, sex, body mass index, genetic predisposition, and dietary patterns; and joint-level factors consisting of previous injury, malalignment, and abnormal joint loading.

Despite its widespread presence and substantial burden, public knowledge and awareness of OA remain inadequate. In Jeddah, Saudi Arabia, Alyami et al. [6], reported substantial gaps in public understanding of OA among 1238 participants, including limited knowledge of its definition, risk factors, diagnosis, and management. Similarly, studies conducted in Makkah, Sudair, Al-Qunfudah, and Hail have reported inadequate awareness and persistent misconceptions regarding OA and its risk factors and management [7–9]. These findings suggest that gaps in public knowledge may persist across different regions of the Kingdom.

Getting information regarding the public knowledge of OA in Makkah and Jeddah will be crucial to formulating effective health education campaigns. By knowing exactly which misconceptions and knowledge gaps exist, interventions can subsequently be tailored accordingly to raise awareness, improve early diagnosis, and promote preventive measures. This is extremely relevant because most of the risk factors of OA, including obesity and not getting proper physical activity, are modifiable [10]. Interaction with healthcare professionals and the launch of educational programs can close this gap and promote a wider understanding of this prevalent condition among the general population. A wider knowledge about osteoarthritis would be achieved if the information is presented in straightforward, easy-to-understand language and provided to the public free of charge [6]. In addition, a study conducted in Hail, Saudi Arabia demonstrated that the level of awareness and knowledge regarding OA among the general population in Hail is deemed inadequate [11].

Despite these studies, limited research has assessed the current level of public knowledge of osteoarthritis across multiple major cities in Saudi Arabia or evaluated whether previously reported knowledge gaps persist in different populations [12]. In particular, while the study by Alyami et al. provides valuable insight into OA awareness among residents of Jeddah, comparable data from neighboring major population centers such as Makkah remain limited [6]. Evaluating public knowledge in both cities and comparing the findings with previously reported data may provide important insights into regional awareness patterns and help determine whether public knowledge has improved over time.

Therefore, this comparative cross-sectional study aims to assess the level of knowledge and awareness regarding osteoarthritis among the general population in Makkah and Jeddah. Additionally, the study seeks to compare the current findings with those reported in previous survey-based studies, particularly the study conducted by Alyami et al. in Jeddah, in order to evaluate current awareness levels and identify persistent misconceptions regarding osteoarthritis risk factors, symptoms, and management. Despite the available Saudi evidence, a key unresolved question is whether the osteoarthritis (OA) knowledge deficits and misconceptions previously documented in Jeddah represent a persistent and geographically broader pattern or have changed over time. The previous Jeddah study demonstrated substantial deficiencies in overall OA knowledge, with recurring misconceptions across disease mechanisms, risk factors, diagnosis, and treatment; however, contemporary evidence evaluating these domains across major Saudi cities remains limited. Accordingly, we hypothesized that the current population of Makkah and Jeddah would demonstrate an overall and item-level OA knowledge profile that differs from that previously reported in Jeddah, while domain specific misconceptions regarding disease mechanisms, risk factors, diagnosis, and treatment would remain evident.

## Methods

### Study design and setting

This study was designed as a comparative cross-sectional survey aimed at assessing the level of public knowledge and awareness regarding OA among the general population residing in the cities of Makkah and Jeddah, Saudi Arabia. The study was conducted between July 2025 and October 2025 following approval from the Institutional Review Board (IRB) of the Ministry of National Guard Health Affairs (NRJ25/055/4) of King Abdullah International Medical Research Center (KAIMRC), Jeddah.

In addition to evaluating the current level of awareness, the results of this study were compared with those reported in a previously published survey conducted among residents of Jeddah to assess whether knowledge patterns and misconceptions regarding osteoarthritis have changed over time.

For this comparison, all data from the previous study by Alyami et al. [6] were obtained directedly from the published article. The extracted data included the reported overall mean knowledge score, mean knowledge scores stratified by demographic and knowledge-related variables, and item-level questionnaire responses. No individual-level data or external dataset from the previous study was accessed or reanalyzed, and no communication with the authors of the previous study for additional data. Therefore, all comparisons were based solely on aggregate results reported in the published article.

### Study population and eligibility criteria

The study targeted adults from the general population living in Makkah and Jeddah. Individuals were eligible to participate if they were 18 years of age or older and able to read Arabic. A previous diagnosis of osteoarthritis was not an exclusion criterion. Participants with and without a personal history of osteoarthritis were therefore eligible to participate, as the study aimed to assess public knowledge and awareness of osteoarthritis across the general adult population.

Participation in the study was voluntary, and informed consent was obtained electronically before respondents were allowed to proceed with the questionnaire.

### Sample size

The required sample size was estimated using the Raosoft^®^ sample size calculator, assuming a 95% confidence level, a 5% margin of error, and a response distribution of 50%. Based on an estimated population of approximately 8,000,000 residents in Makkah and Jeddah, the minimum required sample size was calculated to be 385 participants.

Due to the predefined data collection period, the final sample consisted of 365 completed responses. Although the final sample of 365 participants was slightly below the initially calculated minimum of 385, the achieved sample provided an estimated margin of error of approximately 5.13% at the 95% confidence level, compared with the planned 5.00%. This represents only a minimal reduction in statistical precision.

### Sampling technique

Participants were recruited using a non-probability convenience sampling technique. The survey was distributed electronically through online platforms and social media networks targeting adults residing in Makkah and Jeddah. Current city of residence was self-reported through the questionnaire, with Makkah and Jeddah specified as the eligible study locations. Individuals who met the inclusion criteria and agreed to participate were able to complete the questionnaire anonymously.

### Data collection procedure

**Figure 1.**
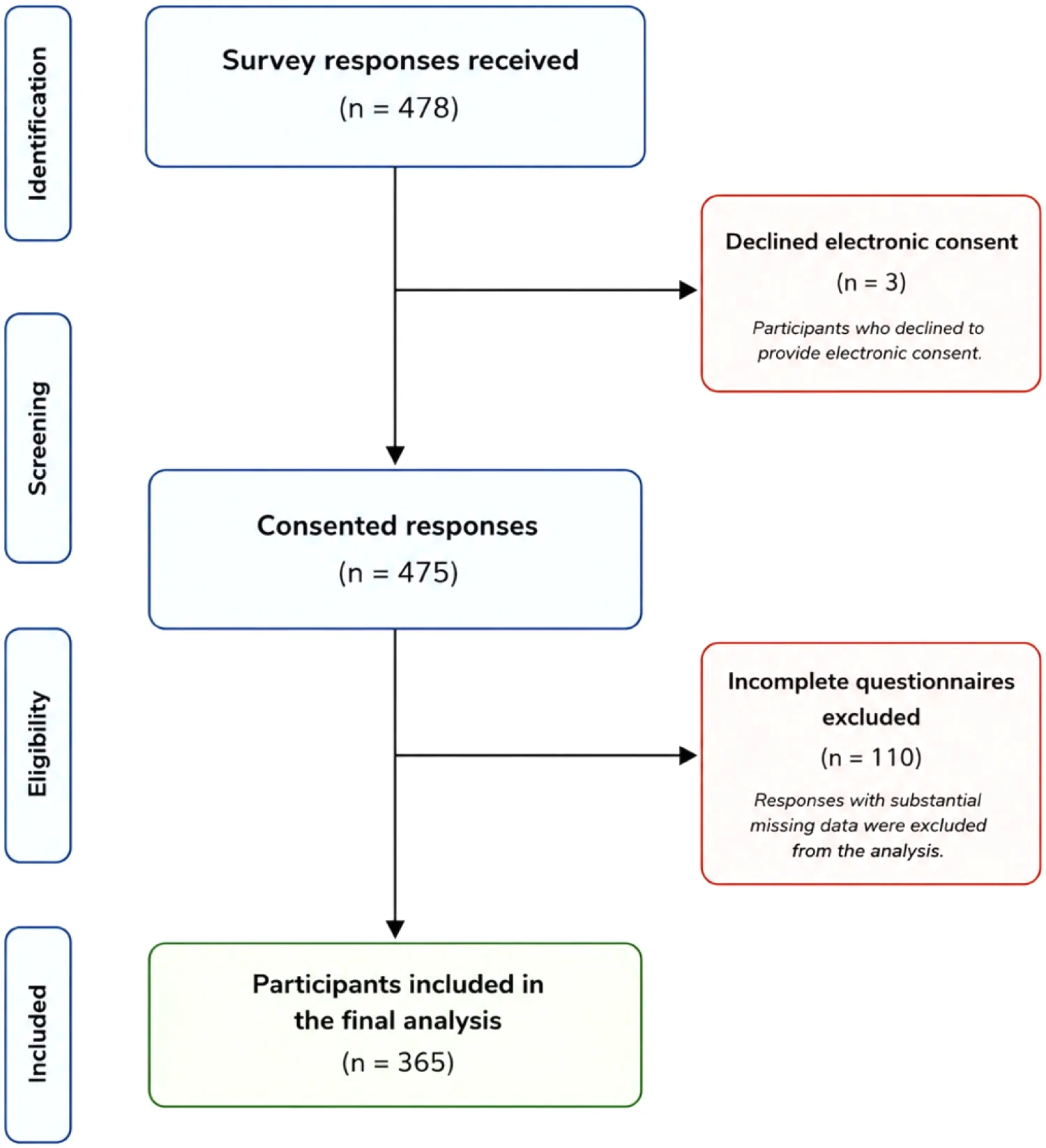
Flow diagram of participant recruitment and inclusion in the final analysis.

Data were collected using a self-administered online questionnaire. Before accessing the survey questions, participants were presented with an electronic informed consent form explaining the study purpose, the voluntary nature of participation, and the confidentiality of responses. Only participants who provided consent were able to proceed to the questionnaire. The online survey platform was configured to allow only one response per account to minimize duplicate submissions. No personally identifiable information was collected, and all responses were recorded anonymously and stored securely. A participant flow diagram illustrating the recruitment and exclusion process is presented in Figure 1.

### Study instrument

Data were collected using the 20-item osteoarthritis knowledge questionnaire adopted from Alyami et al. [6], which was previously used to assess public knowledge of osteoarthritis among residents of Jeddah, Saudi Arabia.

The questionnaire was originally developed following a review of the relevant literature, including adaptation of selected questions from the validated Patient Knowledge Questionnaire for Osteoarthritis (PKQ-OA). In the original study, content validity was assessed by three orthopedic surgeons who reviewed the questionnaire to determine whether the relevant topics were adequately covered and whether any questions were considered irrelevant. Construct and criterion validity were not established because no standardized comparison measure was available.

The original questionnaire was translated from English into Arabic and subsequently back-translated to ensure linguistic consistency. The Arabic version developed through this process was used in the present study without modification; therefore, no additional translation or back-translation was performed.

A pilot study was conducted by Alyami et al. among 25 members of the general public. The pilot participants evaluated the questionnaire with regard to its length, design, wording, comprehensibility, and ease of completion. The pilot participants were not included in the original study sample. As the present study used the same Arabic questionnaire without modification, no separate pilot study was conducted for the present study.

The questionnaire consisted of the following sections:

### Demographic characteristics

Participants were asked to provide demographic information including: City of residence (Makkah or Jeddah), Gender, Age group, Educational level, Whether their academic or professional specialty was related to the medical field.

### Knowledge-related background questions

Participants were asked additional questions regarding: Their understanding of the underlying mechanism of osteoarthritis, their source of information about osteoarthritis, and whether they personally knew someone diagnosed with osteoarthritis.

### Osteoarthritis knowledge assessment

Knowledge regarding osteoarthritis was evaluated using a 20-item questionnaire assessing participants’ understanding of: General facts about osteoarthritis, Symptoms, Risk factors, Diagnostic methods, Treatment options. Each question included three response options: Yes, No, or I do not know. A knowledge score was calculated by assigning one point for each correct answer, resulting in a total possible score ranging from 0 to 20. Higher scores indicated greater knowledge regarding osteoarthritis.

### Reliability

The internal consistency of the 20-item knowledge assessment was evaluated in the present sample using the Kuder–Richardson 20 (KR-20) coefficient because the knowledge items were scored dichotomously as correct or incorrect. The resulting coefficient was reported as a measure of internal consistency for the questionnaire in the present study.

### Methodological comparability with the previous study

The present study was conducted to facilitate comparability with the previous study by Alyami et al. [6], by adopting the same 20-item osteoarthritis knowledge questionnaire and scoring approach. Both studies targeted adults aged ≥18 years from the general population. However, participants with a previous history of osteoarthritis were retained because the study was designed to assess osteoarthritis-related knowledge within the adult population rather than exclusively among individuals without OA. A previous diagnosis of OA does not preclude assessment of disease-related knowledge, and excluding these participants could reduce the representativeness of the sample, particularly because individuals with OA constitute an important segment of the population to whom OA education is relevant. Moreover, the recruitment procedures differed: the previous study recruited participants from a public mall in Jeddah, whereas the present study recruited participants from Makkah and Jeddah through an electronically distributed survey. Although both studies used convenience sampling, differences in recruitment setting, geographic coverage, sample characteristics, and statistical procedures were considered when interpreting comparisons between the two studies.

### Statistical analysis

Data were analyzed using JMP Pro version 19 (SAS Institute Inc., Cary, NC, USA). Descriptive statistics were used to summarize the characteristics of the study population. Continuous variables were presented as means and standard deviations (SD), while categorical variables were expressed as frequencies and percentages.

The mean knowledge score was calculated for the entire sample and across different demographic groups. Differences in mean knowledge scores between two groups were assessed using independent-samples t-tests, while comparisons involving three or more groups were assessed using one-way analysis of variance (ANOVA). Homogeneity of variance was assessed for multi-group comparisons; when this assumption was violated, Welch’s ANOVA was used. Significant omnibus comparisons were followed by Games–Howell post-hoc pairwise testing when unequal variances were present. Comparisons of proportions between categorical variables were evaluated using the chi-square test.

A multivariable linear regression model was performed to identify factors independently associated with total OA knowledge score. The model included city of residence, sex, age group, educational level, medical-field background, and knowing someone diagnosed with osteoarthritis as independent variables. Model performance was summarized using the F statistic, R^2^, and adjusted R^2^. All statistical tests were two-sided, and a *p*-value ≤ 0.05 was considered statistically significant.

### Ethical considerations

The study protocol was reviewed and approved by the Institutional Review Board (IRB) of the Ministry of National Guard Health Affairs (NRJ25/055/4). The study was conducted in full compliance with internationally accepted ethical standards for medical research involving human participants with approval.

Participants were provided with an electronic informed consent form explaining the study objectives, the voluntary nature of participation, and their right to withdraw at any time without consequences. No identifying personal information was collected, and all responses remained anonymous and confidential.

All data were stored on password-protected devices accessible only to authorized members of the research team, and the collected data were used solely for the purposes of this research.

## Results

A total of 365 participants were included, of whom 205 (56.2%) were residents of Jeddah and 160 (43.8%) were residents of Makkah. The majority of participants (54.8%) were male. In terms of age distribution, the majority (39.5%) were 18–29 years old, followed by 17.5% aged 50–59 years, 17.3% aged 30–39 years, and 14.2% aged 40–49 years. Participants aged 60 years or older represented 11.5% of the sample. The 20-item knowledge assessment demonstrated a KR-20 coefficient of 0.70, indicating acceptable internal consistency in the present sample.

The demographic composition differed between the two cities. Gender distribution differed significantly between Jeddah and Makkah (*p* < 0.0001), with females comprising 61.0% of the Jeddah sample compared with 25.0% of the Makkah sample. Age distribution also differed significantly between the two cities (*p* = 0.03), whereas educational level did not differ significantly (*p* = 0.11).

The most frequent educational level was a bachelor’s degree (60.0%), followed by secondary education (24.7%). A smaller proportion (10.4%) achieved higher education, while 4.7% had less than secondary education and 0.3% reported no formal education. The majority of participants (73.7%) reported having a family member or friend diagnosed with osteoarthritis (OA) (Table 1).

**Table 1.** Mean knowledge score by demographic variables.

Item	*N* (%)	Mean knowledge score ± SD	*p*-value
City of residence			**0.52**
Jeddah	205 (56.2)	11.44 ± 3.30	
Makkah	160 (43.8	11.21 ± 3.70	
Gender			**0.97**
Male	200 (54.8)	11.35 ± 3.75	
Female	165 (45.2)	11.33 ± 3.13	
Age			**0.20**
18–29	144 (39.5)	11.86 ± 3.21	
30–39	63 (17.3)	10.89 ± 4.31	
40–49	52 (14.2)	11.33 ± 3.12	
50–59	64 (17.5)	10.97 ± 3.78	
≥60	42 (11.5)	10.81 ± 2.73	
Education			**0.51**
Uneducated‡	1 (0.3)	10.00 ±—	
Less than secondary	17 (4.7)	10.29 ± 3.84	
Secondary	90 (24.7)	11.20 ± 3.34	
Bachelor	219 (60.0)	11.37 ± 3.43	
Higher education	38 (10.4)	12.03 ± 3.94	
Medical-field background			**<0.0001** *
Yes	76 (20.8%)	13.09 ± 2.74	
No	289 (79.2%)	10.88 ± 3.51	

*p-values were obtained using independent-samples t-tests for two-group comparisons and one-way analysis of variance (ANOVA) for comparisons involving three or more groups*.

* *p* ≤* 0.05 was considered statistically significant*.

^‡^ *SD could not be calculated for the uneducated category because it contained only one participant (n = 1)*.

*There were no missing data for the variables presented in* Table 1.

Approximately 61.4% of participants were knowledgeable about the underlying mechanism of OA, correctly identifying it as a process in which the protective cartilage at the bone ends wears down over time. A smaller proportion (3.3%) indicated acid accumulation inside the joint, while 11.0% believed that OA is caused by compression of a nerve passing near the joint. It is noteworthy that 17.8% reported not knowing the mechanism of OA at all.

In terms of source of information, the most common source was relatives and friends (35.9%), followed by the internet and social media (27.1%). A smaller proportion obtained information from school or university (17.5%), personal experience (9.9%), or physicians (6.3%) (Table 2). The mean score for the 20-item knowledge quiz was 11.34 ± 3.48. The mean knowledge score was 11.44 ± 3.30 among participants from Jeddah and 11.21 ± 3.70 among participants from Makkah, with no statistically significant difference between the two cities (*p* = 0.52).

**Table 2.** Knowledge by knowledge-related responses and source of information.

Item	*N* (%)	Mean knowledge score ± SD	*p*-value
Underlying mechanism			**<0.0001** *
Cartilage wears down over time	224 (61.4)	12.29 ± 3.02	
Nerve compression near joint	40 (11.0)	10.95 ± 2.87	
Decreased blood supply with aging	24 (6.6)	10.50 ± 2.92	
Acid accumulation in joint	12 (3.3)	10.58 ± 2.97	
I don’t know	65 (17.8)	8.77 ± 4.14	
Source of information			**<0.0001** *
Personal experience	36 (9.9)	11.39 ± 3.50	
Relatives and friends	131 (35.9)	10.56 ± 3.62	
Television	4 (1.1)	8.00 ± 5.42	
Internet and social media	99 (27.1)	11.22 ± 3.50	
School or university	64 (17.5)	13.33 ± 2.77	
Books / magazines / newspapers	8 (2.2)	12.50 ± 2.20	
Physician	23 (6.3)	10.83 ± 3.70	
Knowing patient with oa			**0.0011** *
Yes	269 (73.7)	11.69 ± 3.30	
No	96 (26.3)	10.35 ± 3.77	

*p-values were obtained using one-way ANOVA, Welch’s ANOVA, or independent-samples t-test, as appropriate. Categories with small sample sizes, particularly television (n = 4) and books/magazines/newspapers (n = 8), should be interpreted with caution*.

* *p* ≤* 0.05 was considered statistically significant. SD, standard deviation; OA, osteoarthritis*.

Regarding individual questionnaire responses, the distribution of participants’ sources of OA information differed significantly between Jeddah and Makkah (*p* = 0.03). Relatives and friends were reported as the source of information by 41.0% of participants in Jeddah compared with 29.4% in Makkah, whereas school or university was reported by 13.7% and 22.5%, respectively. In contrast, responses regarding the underlying mechanism of OA did not differ significantly between the two cities (*p* = 0.83). Among the individual knowledge items, a significant difference between the two cities was observed only for the item concerning microorganisms as a possible cause of OA (*p* = 0.04). A higher proportion of participants from Makkah answered “Yes” compared with those from Jeddah (25.6% vs. 15.1%, respectively). No statistically significant differences were observed for the remaining knowledge items.

The first part of the quiz consisted of 5 general facts regarding OA. In terms of it being a chronic problem, 55.9% responded yes, and 86.8% did not think OA was a rare disease. The majority (81.9%) knew that OA can affect different joints. However, 26.3% correctly responded that OA is not caused by cold, damp weather, and 38.6% correctly indicated that OA is not caused by a microorganism.

The second part of the quiz included 4 questions about the main symptoms of OA. More than half (57.8%) knew that pain is not the only symptom, 58.9% indicated stiffness, and 57.0% knew that swelling is a sign of OA. The majority (68.8%) agreed that OA could lead to loss of joint movement.

The third part assessed knowledge about the main risk factors of OA. 42.7% of participants knew that genetic factors may predispose individuals to OA, while the majority (91.2%) recognized aging as an important risk factor. Regarding gender differences, 51.0% indicated that men and women are not equally affected by OA.

The last part of the quiz focused on the diagnosis and treatment of OA. Approximately 69.0% of participants indicated that physical examination and X-ray imaging are used to diagnose OA, while 46.8% correctly indicated that blood tests are not used for diagnosis. Regarding treatment, 53.2% reported that non-steroidal anti-inflammatory drugs (NSAIDs) improve OA symptoms. The majority (69.0%) found that exercise such as swimming is beneficial, and 82.5% believed that physiotherapy could improve OA symptoms. However, 22.7% believed that acid-free diets are a proven treatment, and 46.3% believed that intra-articular injections such as stem cells or hyaluronic acid could cure OA. Finally, 57.5% indicated that joint replacement surgery is the ultimate option to relieve OA symptoms (Table 3).

**Table 3.** Participants’ responses to the 20-item OA knowledge quiz.

Item	Yes *n* (%)	No *n* (%)	Not sure *n* (%)	Correct answer
OA is a chronic disease	**204 (55.9)**	77 (21.1)	84 (23.0)	Yes
OA is rare	15 (4.1)	**317 (86.8)**	33 (9.0)	No
OA can affect different joints	**299 (81.9)**	23 (6.3)	43 (11.8)	Yes
Cold weather causes OA	137 (37.5)	**96 (26.3)**	132 (36.2)	No
Microorganisms cause OA	72 (19.7)	**141 (38.6)**	152 (41.6)	No
Pain is the only symptom	89 (24.4)	**211 (57.8)**	65 (17.8)	No
Stiffness is a symptom	**215 (58.9)**	35 (9.6)	115 (31.5)	Yes
Swelling is a sign	**208 (57.0)**	61 (16.7)	96 (26.3)	Yes
OA can lead to loss of joint movement	**251 (68.8)**	45 (12.3)	69 (18.9)	Yes
Genetic factors predispose to OA	**156 (42.7)**	122 (33.4)	87 (23.8)	Yes
Aging is a risk factor	**333 (91.2)**	10 (2.7)	22 (6.0)	Yes
Men and women equally affected	107 (29.3)	**186 (51.0)**	72 (19.7)	No
Physical exam and x-ray diagnose OA	**252 (69.0)**	39 (10.7)	74 (20.3)	Yes
Blood tests diagnose OA	74 (20.3)	**171 (46.8)**	120 (32.9)	No
Nsaid improve OA symptoms	**194 (53.2)**	67 (18.4)	101 (27.7)	Yes
Exercise such as swimming is beneficial	**252 (69.0)**	48 (13.2)	65 (17.8)	Yes
Acid-free diets treat OA	83 (22.7)	**51 (14.0)**	231 (63.3)	No
Physiotherapy improves OA symptoms	**301 (82.5)**	22 (6.0)	42 (11.5)	Yes
Stem cell / hyaluronic injections cure OA	169 (46.3)	**42 (11.5)**	154 (42.2)	No
Joint replacement is final treatment option	**210 (57.5)**	62 (17.0)	93 (25.5)	Yes

OA, osteoarthritis; NSAIDs, non-steroidal anti-inflammatory drugs. Correct answers were determined according to the scoring key of the original validated questionnaire.

When comparing the mean score based on gender, the male subgroup scored 11.35 ± 3.75 and the female subgroup scored 11.33 ± 3.13, with no statistically significant difference (*p* = 0.97). Among the age groups, participants aged 18–29 years had the highest mean knowledge score (11.86 ± 3.21); however, the difference across age groups was not statistically significant (*p* = 0.20).

Participants with higher education had the highest mean knowledge score (12.03 ± 3.94), whereas those with less than secondary education had a mean score of 10.29 ± 3.84. However, differences in knowledge scores across educational levels were not statistically significant (*p* = 0.51). Participants with a medical-field background had a significantly higher mean knowledge score than those without a medical-field background (13.09 ± 2.74 vs. 10.88 ± 3.51, respectively; *p* < 0.0001).

In terms of responses related to the underlying mechanism of OA, the group that correctly identified the mechanism scored 12.29 ± 3.02, which was significantly higher than the other groups (*p* < 0.001). Participants who indicated school or university as their source of information scored 13.33 ± 2.77, which was also significantly higher than other sources (*p* < 0.001). In addition, participants who knew someone diagnosed with OA had a significantly higher mean score (11.69 ± 3.30) compared with those who did not (10.35 ± 3.77, *p* = 0.0011).

In the multivariable linear regression model (*n* = 365), medical-field background and knowing someone with OA were independently associated with higher knowledge scores (both *p* < 0.0001), whereas city of residence (*p* = 0.26), sex (*p* = 0.90), age (overall *p* = 0.70), and educational level (overall *p* = 0.19) were not significantly associated with knowledge score. The overall model was statistically significant (*F* = 4.39, *p* < 0.0001), with an R^2^ of 0.130 and an adjusted R^2^ of 0.101 (Table 4).

**Table 4.** Multivariable linear regression analysis of factors associated with total osteoarthritis knowledge score.

PREDICTOR	B coefficient	95% CI	*p*-value
City of residence			**0.26**†
Jeddah	0.220	−0.160 to 0.599	0.26
Gender			**0.90**†
Female	0.023	−0.351 to 0.397	0.90
Age			**0.70**†
18–29	0.408	−0.286 to 1.102	0.25
30–39	−0.330	−1.083 to 0.423	0.39
40–49	0.188	−0.625 to 1.000	0.65
50–59	—‡	—‡	—‡
≥60	−0.130	−1.021 to 0.761	0.78
Education			**0.19**†
Uneducated‡	−2.839	−8.093 to 2.414	0.29
Less than secondary	—‡	—‡	—‡
Secondary	0.434	−1.043 to 1.911	0.56
Bachelor	0.931	−0.486 to 2.347	0.20
Higher education	1.678	0.039 to 3.317	0.045*
Medical-field background			**<0.0001** *
No	−1.157	−1.650 to −0.665	<0.0001*
Knowing someone diagnosed with OA			**<0.0001** *
No	−0.838	−1.245 to −0.432	<0.0001*

* *B, unstandardized regression coefficient; CI, confidence interval; OA, osteoarthritis*.

^†^ *p-values shown for the main predictor rows represent overall effects from the model Effect Tests*.

^‡^ *The category was included in the model but is not displayed as a separate parameter estimate because categorical predictors were parameterized using effect coding in JMP. p* ≤* 0.05 was considered statistically significant. The overall model was statistically significant, F(12,352) = 4.392, p < 0.0001, R^2^ = 0.130, adjusted R^2^ = 0.101 (n = 365)*.

## Discussion

This study aimed to evaluate the general population knowledge about OA and its related risk factors in Makkah and Jeddah cities in Saudi Arabia in comparison to the previous study that was published in 2020 [6]. A higher proportion of participants were from Jeddah, with 205 respondents constituting 56.2% of the total sample, compared with 160 respondents (43.8%) from Makkah. Unlike the previous study which had the females as the majority of the participants [6]. The gender distribution of this study showed that the majority of the participants were males (54.8%). Moreover, more than a third of the study sample were between 18 and 29 years old (39.5%). This percentage is less than the previous study which was done in 2020 and showed that (52%) were between the ages of 18 and 29 [6]. However, the difference could be explained by the nature of the sampling method which is the electronic survey. Similar to the previous study, approximately two thirds of the participants held a bachelor’s degree (60%) [6]. Furthermore, the majority of participants did not have a medical-field background (79.2%).

More than half of the participants knew that OA is caused by progressive degeneration of the articular cartilages (61.4%) in comparison to the previous study which reported that only 37% knew the mechanism of OA [6]. However, a similar proportion of the sample in both this study and the previous one showed that (17.8%) and (15.3%) did not know the mechanism behind OA respectively [6]. Regarding the source of information (35.9%) knew about OA from a relative or a friend and (27.1%) knew about it from social media. This shows that approximately two thirds of the sample did not have a reliable source of information with only (6.3%) acquired their knowledge from a doctor. Similarly, the previous article shows comparable percentages regarding that they knew about OA from a relative or a friend (44.8%) and that only (7.6%) of the sample knew about OA from a physician [6]. On the other hand, the percentage of people who knew about OA from the internet or social media was higher in this sample compared to the previous article (19.2%) [6]. Health misinformation is known to be disseminated through social media platforms [13]. However, although internet and social media were more frequently reported as sources of OA information in the present study than in the previous survey, the present study did not assess the accuracy or quality of information obtained from these sources. Therefore, no inference can be made regarding whether these sources contributed to the misconceptions observed among participants. The overwhelming majority knew someone with OA (73.7%) this is most likely due to the high prevalence of OA among the Saudi population which one systematic review and meta analysis reported patients who are older than 40 years of age had a prevalence of OA as high as 67.8% on average [14].

More than half of the participants knew that OA is a chronic disease (55.9%), yet this percentage is lower than the percentage in the previous study (67.5%) [6]. Moreover, most of the participants knew that OA is not a rare disease (86.8%). In contrast to the previous study which showed that only (36.6%) knew that OA could affect different joints, (81.9%) of the participants in this study knew that OA could affect different joints [6]. Similar to the number reported in the previous study (42%), a considerable number of participants had the misconception that OA could be caused by cold or damp weather (41.6%) [6]. Surprisingly, (41.6%) did not know if microorganisms could cause OA or not, and only (38.6%) knew that microorganisms are not related to OA. This number is comparable to the one reported in the previous study which showed that only (38.5%) knew that OA is not due to microorganisms [6].

### Symptoms of OA

More than 60% of participants were aware that the pain is not the only symptom of OA. This reflects the better knowledge of the public about this disease, as there are other symptoms that worsens the quality of life, like limitations of range of motion [15]. In addition, this survey showed that joint stiffness, swelling, and reduced range of motion are among the most common symptoms recognized by the participants. Joint stiffness reported by 58.9% which was higher than the previous survey, which was published in 2020, which demonstrates that only 51.3% reported joint stiffness [6]. In addition, 57% of the participants mentioned swelling as a common symptom. This symptom was reported less in the last survey by 40% [6]. Regarding range of motion the result of this study showed 68.8%. It was higher than the last survey that mentioned 67% [6].

### Risk factors of OA

More than 90% of this study participants reported that the disease is associated with advanced age. This may show that advanced age is the strongest risk factor for developing this disease [16]. These results correlate with the previous survey which also demonstrated that more than 80% of their participants knew that age is a risk factor for OA [6]. Moreover, genetic predisposition plays an important role for developing OA [17]. This survey showed that about 42.7% of all participants knew that genetic predisposition may be associated with higher risk of developing OA. The last survey showed only 37.3% of the participants know about the genetic predisposition [6]. This can reflect the increase of the awareness of the general people. In addition, more than 50% of participants knew that OA did not equally affect both genders with a higher change of developing OA among female patients.

### Diagnosis and treatment of osteoarthritis

To diagnose OA the main components are imaging, history and physical examination [18]. The value of blood biochemical markers in diagnosing OA remains limited [19]. This study showed that 69% of all participants knew that physical examination and x-ray are used for the diagnosis of OA which reflects a good level of knowledge among public people about the diagnosis of OA. This data is contradictory with the previous survey that showed less than 50% of participants understand that the diagnosis depends on physical examination and x-ray [6]. This may demonstrate the better awareness of public people about OA. In addition, there is an obvious improvement in the general awareness regarding the limited benefit of getting blood tests for the diagnosis of OA with 46.8% in this survey compared to the previous one which represents only 42% of all participants [6].

Regarding the treatment of OA, our survey found that 53.2% of participants knew that non-steroidal anti-inflammatory drugs (NSAIDs) can reduce the symptoms of OA. This percentage was higher than the previous survey where only 46% of participants recognized that NSAIDs can help control the symptoms of OA [6]. The proportion of participants regarding the benefits of exercise in relieving the symptoms of OA were the same in both surveys, with a percentage of 69% in each survey reporting that exercise such as swimming can help in controlling the symptoms of OA [6]. This could reflect that more than half of the public are aware of the benefits of exercise as a treatment option of OA. One indicator of improvement was noticed in this survey is that only 22.7% of participants believed that acid-free diets could treat OA compared to the last published survey where more than half of participants agreed that acid-free diets may treat OA [6]. This result reflects a better level of awareness in the public regarding misconceptions about OA treatment. Physiotherapy is considered one of the options in treatment of OA as it improves the function and the overall wellbeing of the patients [20]. In this study, 82.5% of participants reported that physiotherapy could significantly improve symptoms of OA. In comparison, in the previous study, 75% of participants found that physiotherapy can improve OA [6]. This reflects the better understanding of the importance of non-pharmacological treatment, such as physiotherapy as treatment of OA. In the present study, 46.3% of participants believed that stem-cell or hyaluronic acid injections could cure OA, representing a misconception according to the questionnaire scoring key. This proportion was higher than the 28% reported in the previous survey for the corresponding injection-related item [6]. Although the previous study discussed the potential symptomatic benefits of intra-articular hyaluronic acid therapy, such benefits should be distinguished from curing OA. Therefore, the higher proportion observed in the present study should not be interpreted as improved knowledge, but rather as persistence of a misconception regarding the role of these injections in OA treatment. More than half of participants knew that joint replacement surgery is the final treatment option of OA. This percentage was higher than the previous survey which found only 35% of participants agreed that joint replacement surgery is the final treatment option of OA.

### Participant scores stratified by gender, age, level of education, underlying mechanism, source of information and knowing someone with osteoarthritis

This survey used a scoring system giving each correct answer a point out of a total of 20 points. The overall mean score was 11.34 ± 3.48, which was higher than the mean score of 9.84 reported in the previous study [6]. In unadjusted analyses, knowledge scores did not differ significantly according to gender (*p* = 0.97), educational level (*p* = 0.51), or age (*p* = 0.20). These associations also remained nonsignificant after multivariable adjustment for sex (*p* = 0.90), educational level (overall *p* = 0.19), and age (overall *p* = 0.70). Although participants with higher education had the highest unadjusted mean knowledge score (12.03 ± 3.94), the overall difference across educational levels was not statistically significant. These findings differ partly from the previous study, in which educational level was significantly associated with knowledge score [6]. Participants with a medical-field background had higher knowledge scores, and this association remained statistically significant after adjustment for city of residence, sex, age, educational level, and familiarity with an individual diagnosed with osteoarthritis (*p* < 0.0001).

Regarding knowledge of the underlying mechanism of OA, participants who correctly identified the mechanism had the highest mean OA knowledge score in both studies; however, the mean score was higher in the present study (12.29 ± 3.02) compared with 10.67 in the previous study [6]. In both studies, participants who acquired their knowledge from school or university had the highest mean OA knowledge score, with 10.38 in the previous study and 13.33 ± 2.77 in the present study [6]. Participants who reported books, magazines, or newspapers as their source of information had a mean OA knowledge score of 12.50 ± 2.20 in the present study, compared with 8.90 in the previous study [6]. However, estimates for less frequently reported information sources, particularly television (*n* = 4) and books, magazines, or newspapers (*n* = 8), should be interpreted cautiously because of the small number of participants in these categories.

Lastly, participants who knew someone diagnosed with OA had a significantly higher mean knowledge score than those who did not (11.69 ± 3.30 vs. 10.35 ± 3.77, respectively; *p* = 0.0011). This association remained statistically significant after multivariable adjustment (*p* < 0.0001), suggesting that familiarity with an affected individual may contribute to greater awareness and knowledge of OA.

### Comparison with other studies conducted in Saudi Arabia

The findings of the present study are broadly consistent with previous research conducted in different regions of Saudi Arabia, which collectively indicates that public awareness of osteoarthritis (OA) remains suboptimal and is often accompanied by persistent misconceptions regarding its causes and management. In the current study, the mean knowledge score was 11.34 ± 3.48, reflecting substantial remaining gaps in OA knowledge among residents of Makkah and Jeddah. Similar patterns have been reported in other Saudi regions. For example, a study conducted in Makkah found that only 35.8% of participants demonstrated adequate awareness of OA, highlighting substantial knowledge gaps among the general population [7]. Comparable findings were reported in the Sudair region, where the majority of respondents had heard of osteoarthritis but lacked detailed understanding of its symptoms, risk factors, and preventive measures [8]. Likewise, a research conducted in Al-Qunfudah concluded that the public’s knowledge regarding OA remained limited and inadequate [9].

Similar concerns regarding insufficient public awareness were also documented in northern Saudi Arabia. A study in the Hail region reported that the level of knowledge regarding OA among the general population was inadequate, emphasizing the need for targeted health education initiatives [11]. On the other hand, The general population in the Aseer region demonstrated a high level of awareness regarding knee osteoarthritis, particularly in relation to preventive strategies, symptom-relieving measures, and associated risk factors [12]. When viewed collectively, these findings suggest that gaps in OA awareness are not limited to a single geographic area but appear to represent a nationwide public health knowledge gap across multiple regions of Saudi Arabia.

### Clinical and public health implications

The present study contributes to the existing literature by providing updated evidence on osteoarthritis knowledge across two major Saudi cities and by identifying knowledge gaps that remain despite differences observed in overall awareness compared with previous study. Beyond describing the level of public knowledge, the findings identified medical-field background and familiarity with an individual diagnosed with osteoarthritis as factors independently associated with greater OA knowledge. These findings may help guide the development of targeted health education strategies that focus on correcting misconceptions regarding osteoarthritis risk factors, diagnosis, and treatment. From a public health perspective, improving access to reliable and evidence-based information may support greater understanding of osteoarthritis, encourage appropriate preventive behaviours, and reduce reliance on potentially inaccurate sources of health information.

### Strengths and limitations

This study has several strengths. First, it assessed osteoarthritis awareness in two major cities in Saudi Arabia, which adds broader regional value compared with studies limited to a single city. Second, the study used a previously validated questionnaire adopted from an earlier published study, allowing meaningful comparison with prior findings and helping evaluate whether awareness patterns have changed over time. Third, the questionnaire covered multiple important domains of osteoarthritis knowledge, including general facts, symptoms, risk factors, diagnosis, and treatment, providing a comprehensive assessment of public awareness. Additionally, because the survey was distributed electronically through online platforms and social media, the total number of individuals who received or viewed the survey invitation could not be determined; therefore, a conventional response rate could not be calculated. Finally, the study identified medical-field background and familiarity with an individual diagnosed with osteoarthritis as factors independently associated with greater knowledge after multivariable adjustment, which may help guide future awareness campaigns and health education strategies.

This study also has several limitations. First, the use of a cross-sectional design limits the ability to determine causality between participant characteristics and knowledge level. Second, the study relied on convenience sampling through an online survey, which may have introduced selection bias and may limit the generalizability of the findings to the wider population of Makkah and Jeddah, particularly older adults or individuals with limited internet access. Furthermore, the sample included a high proportion of participants with university-level education, which may limit the representativeness of the findings and could result in overestimation of OA knowledge in the general population. Third, although the calculated minimum sample size was 385 participants, the final sample included 365 respondents, which was slightly lower than the target. Fourth, the questionnaire depended on self-reported responses, which may be affected by recall bias or social desirability bias. Additionally, no validated cutoff was available to categorize the overall knowledge score as poor, moderate, or good; therefore, descriptions of the observed knowledge level should be interpreted cautiously. Furthermore, multiple unadjusted statistical comparisons were performed without correction for multiplicity, which may increase the risk of type I error; therefore, isolated statistically significant findings should be interpreted cautiously. Finally, while comparison with prior studies is valuable, differences in sampling methods, participant characteristics, and timing between studies should be considered when interpreting changes in awareness over time.

## Conclusion

Compared with several of these regional studies, the present study demonstrates relatively improved awareness in certain areas, particularly regarding the role of aging as a risk factor and the benefits of exercise and physiotherapy in symptom management. Internet and social media were reported as a source of OA information by a substantial proportion of participants; however, the present study did not assess the quality or accuracy of information obtained from these sources or establish their relationship with specific misconceptions. Therefore, no causal inference can be made regarding the influence of digital or social-media information on participants’ OA knowledge. Therefore, the findings reinforce the importance of structured public health education programs and improved dissemination of reliable medical information to enhance public understanding of osteoarthritis and promote preventive strategies across Saudi Arabia.

## Data Availability

Data are available from the corresponding author upon reasonable request.
